# Life habits, hox genes, and affinities of a 311 million-year-old holometabolan larva

**DOI:** 10.1186/s12862-015-0428-8

**Published:** 2015-09-29

**Authors:** Joachim T. Haug, Conrad C. Labandeira, Jorge A. Santiago-Blay, Carolin Haug, Susan Brown

**Affiliations:** Ludwig Maximilians University Munich, Biocenter – Department of Biology II and GeoBio-Center, Großhaderner Str. 2, Planegg-Martinsried, 82152 Germany; Department of Paleobiology, National Museum of Natural History, Smithsonian Institution, Washington DC, 20013 USA; Department of Entomology, University of Maryland, College Park, MD 20742 USA; College of Life Sciences, Capital Normal University, Beijing, 100048 China; Department of Crop and Agroenvironmental Sciences, University of Puerto Rico, Mayagüez, PR 00681 USA; Division of Biology, Kansas State University, Manhattan, KS 66502 USA

**Keywords:** *Abdominal-A*, Caterpillar, *Distalless*, Endopterygote, Eruciform, Late Carboniferous, Mazon Creek, Mouthparts, Trunk appendages, *Ultrabithorax*

## Abstract

**Background:**

Holometabolous insects are the most diverse, speciose and ubiquitous group of multicellular organisms in terrestrial and freshwater ecosystems. The enormous evolutionary and ecological success of Holometabola has been attributed to their unique postembryonic life phases in which nonreproductive and wingless larvae differ significantly in morphology and life habits from their reproductive and mostly winged adults, separated by a resting stage, the pupa. Little is known of the evolutionary developmental mechanisms that produced the holometabolous larval condition and their Paleozoic origin based on fossils and phylogeny.

**Results:**

We provide a detailed anatomic description of a 311 million-year-old specimen, the oldest known holometabolous larva, from the Mazon Creek deposits of Illinois, U.S.A. The *head* is ovoidal, downwardly oriented, broadly attached to the anterior thorax, and bears possible simple eyes and antennae with insertions encircled by molting sutures; other sutures are present but often indistinct. Mouthparts are generalized, consisting of five recognizable segments: a clypeo-labral complex, mandibles, possible hypopharynx, a maxilla bearing indistinct palp-like appendages, and labium. Distinctive mandibles are robust, triangular, and dicondylic. The *thorax* is delineated into three, nonoverlapping regions of distinctive surface texture, each with legs of seven elements, the terminal-most bearing paired claws. The *abdomen* has ten segments deployed in register with overlapping tergites; the penultimate segment bears a paired, cercus-like structure. The anterior eight segments bear clawless leglets more diminutive than the thoracic legs in length and cross-sectional diameter, and inserted more ventrolaterally than ventrally on the abdominal sidewall.

**Conclusions:**

*Srokalarva berthei* occurred in an evolutionary developmental context likely responsible for the early macroevolutionary success of holometabolous insects. *Srokalarva berthei* bore head and prothoracic structures, leglet series on successive abdominal segments – in addition to comparable features on a second taxon eight million-years-younger – that indicates Hox-gene regulation of segmental and appendage patterning among earliest Holometabola. *Srokalarva berthei* body features suggest a caterpillar-like body plan and head structures indicating herbivory consistent with known, contemporaneous insect feeding damage on seed plants. Taxonomic resolution places *Srokalarva berthei* as an extinct lineage, apparently possessing features closer to neuropteroid than other holometabolous lineages.

**Electronic supplementary material:**

The online version of this article (doi:10.1186/s12862-015-0428-8) contains supplementary material, which is available to authorized users.

## Background

**Fig. 1:**
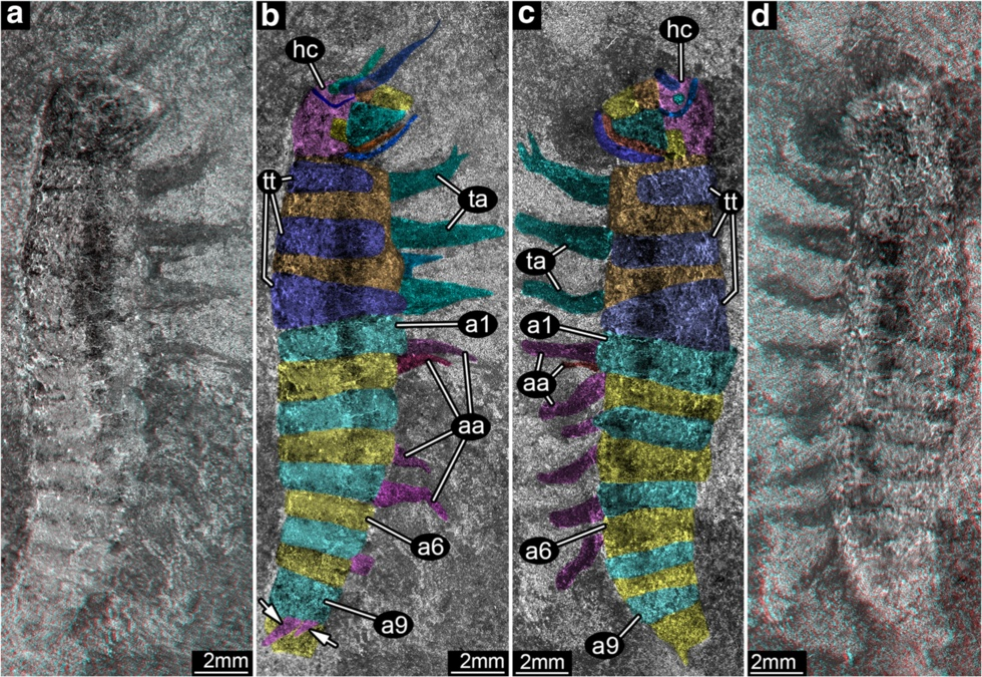
The external body structure of *Srokalarva berthei*. **a** and **b** are the part of specimen MCP-322; (**c**) and (**d**) are the counterpart to MCP-322, representing approximate mirror images of each other. **a** and **d**, Red–cyan stereo anaglyphs imaged under cross-polarized light. (For best visual results, use red–cyan glasses for viewing.) (**b**) and (**c**), Interpretative versions of (**a**) and (**d**), respectively. Colors delimit regions of the head, tergites, thoracic membranous regions, and appendages such as mouthparts, thoracic legs and abdominal prolegs. Arrows indicate abbreviated appendages on abdominal segment 9. Note that the proximal regions of the appendages are concealed under the body, and are not clearly marked. Abbreviations: **aa**, abdominal appendages; **a1**–**a9**, abdominal segments 1 to 9; **hc**, head capsule; **ta**, thoracic appendages; **tt**, trunk tergites

Although holometabolous (endopterygote) insects are the pre-eminent macroscopic animals that structure terrestrial ecosystems today [1], very little is known of their late Paleozoic origins when life on land was overwhelmingly dominated by nonholometabolous insects and a myriad of other arthropod lineages [2]. Various proposals have been offered to explain the evolutionary transition from non-holometabolous to holometabolous in insects [2–4]. Although these evolutionary developmental mechanisms have been addressed by evidence from modern model organisms [3, 4], there is minimal data from the deep-time fossil record regarding the origin of this unique developmental innovation. The Paleozoic fossil record of holometabolous larvae is extremely sparse [6–8], particularly for the time interval during which crucial developments likely occurred [9, 10]. One recent discovery is a holometabolous larva from the Late Pennsylvanian Period of Germany, *Metabolarva bella* [8]. However, insufficient structural details are known about this larva and it remains unplaced in an evolutionary developmental context that would lend understanding of how holometaboly originated. The only other early fossil of a presumptive holometabolous larva is from the older Middle Pennsylvanian Period, *Srokalarva berthei* (Fig. 1, Additional file 1: Figures S1 and S2), an informal designation provided by Kukalová-Peck [6]. The holometabolous identity of the single specimen of *Srokalarva berthei* repeatedly has been questioned [11–13], but without examination of the fossil (see Additional file 1 for a taxonomic assessment). We present a detailed morphological description of *Srokalarva berthei* based on current imaging techniques (Additional file 1), and indicate its significance for early evolution of Holometabola.

## Results

We provide additional documentation and a formal description of *Srokalarva berthei* morphology in the Additional file 1, where important structural details on a tagma-by-tagma and segment-by-segment basis are listed and described. Relevant details regarding the taxonomic affiliation of this specimen with particular early-appearing lineages of Holometabola also are provided.

### The head and mouthparts

The head is less well-preserved than previously thought. In the original reconstruction, head structures [6, 14] were divided into substructures such as cephalic capsule sclerites, antennal articles and palpal segments. Under certain angles of illumination weak subdivisions of these structures are apparent, but other angles of illumination reveal alternative demarcations. Neither surface texture or ornamentation, nor color provides a clear guide for the presence of cephalic sutures (Additional file 1). It is likely that the cephalic capsule indeed was subdivided, yet their exact delimitation remains indeterminate. Fundamental head structures are evident, such as the antennae, clypeus and its surrounding sutures, some primary segments, and the mouthparts and their subelements.

**Fig. 2:**
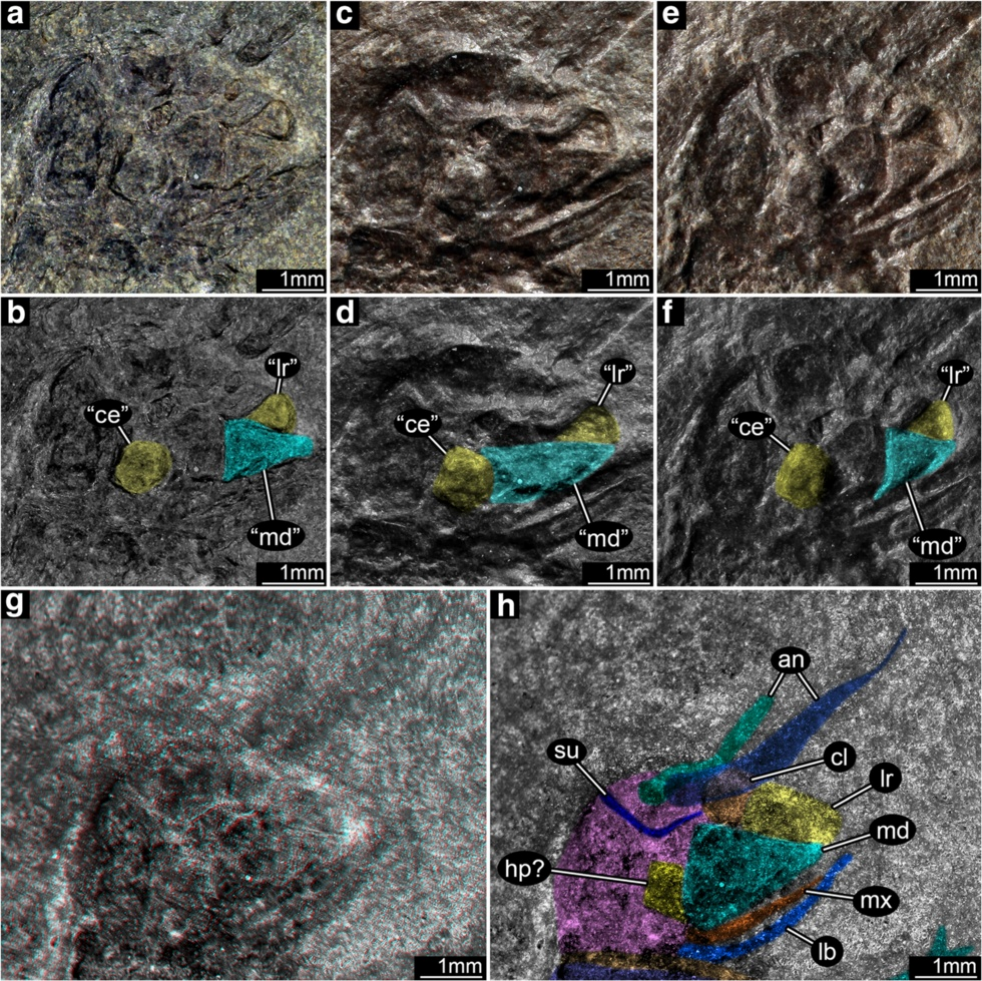
Head structures of *Srokalarva berthei* and some derived artifacts. **a**, Unpolarized, reflected light. **b**, An interpretative version of (**a**). **c** and **e**, Low angle unpolarized light, from multiple source directions. **d** and **f**, Interpretative versions of C and E. **g**, Red–cyan stereo anaglyph under cross-polarized light. (For best visual results, use red–cyan glasses for viewing.) **h**, Interpretative version of (**g**). Note how the mandible shape depends on variation in lighting. Only the stereo anaglyph provides a neutral evaluation of head structure. Abbreviations: **an**, antenna; “**ce**”, presumptive compound eye; **cl**, clypeus; **hp?**, possible hypopharynx of the intercalary segment; **lb**, labium; “**lr**”, labrum interpreted by Kukalová-Peck [5]; **lr**, labrum interpreted by us; “**md**”, mandible interpreted by Kukalová-Peck (1997); **md**, mandible interpreted by us; **mx**, maxilla; **su**, suture, possibly the epicranial suture

Two darker spots positioned anterodorsally most likely represent antennal insertions. Impressions of the more distal parts of the elongate, filiform antennae appear to be directed toward these dark spots, one antenna of which overlies the proximal clypeal region. An additional indication that these spots are antennal insertions is presence of an encircling ridge that originally was interpreted as a molting suture. Anatomically below the antennal insertions and anterior to the mandible is the clypeo-labral complex which bears well-resolved surface texture and a suture that likely was articulatory (Fig. 2g, h). Under cross-polarized light the clypeus is shorter and is inserted further dorsally whereas the labrum appears significantly longer than originally reconstructed (Fig. 2a–f).

The most prominent mouthpart element is a large, triangular mandible, identifiable by shape and position. The mandible is ill defined and the inner surface apparently has partly collapsed while the outer border has remained intact. Our interpretation of the mandible is a more massive structure than provided in the initial account [6]. The mandible under reflected light from above (Fig. 2a, b) or at other angles of incidence (Fig. 2c, d) would suggest a surface bounded by a proximal border, indicating a less massive structure. However, at different light angles (Fig. 2e, f), the proximal border appears positioned further dorsal and the general shape is more slender. The expanded size of the mandible is corroborated by stereomicroscopic imaging (Fig. 2g, h). Above the dorsal mandible margin is a structure more challenging to interpret. This structure is broad, lobate, well-sclerotized and originally was interpreted as an eye, possibly compound. This structure is prominently upraised (Fig. 2), but with no indication it contains ocular features, and appears to have been partly compressed under the mandible. These features suggest that it is related to the mouthparts; the most plausible interpretation is a hypopharynx. A less likely possibility is that it represents a large, projecting condyle of the opposite mandible. (Although it appears the specimen lacks compound eyes, a cluster of miniscule, circular structures may be stemmata (Fig. 1a–f), but their identity is ambiguous.) The two serial structures that are posterior to the mandible (Fig. 2g, h) likely represent head segmental regions with ventral appendages. Based on structure and position, they are interpreted as the maxilla and labium, as originally described. A suture separating these two, posterior, segmental regions from the rest of the head capsule was not observed.

### The thorax and legs

**Fig. 3:**
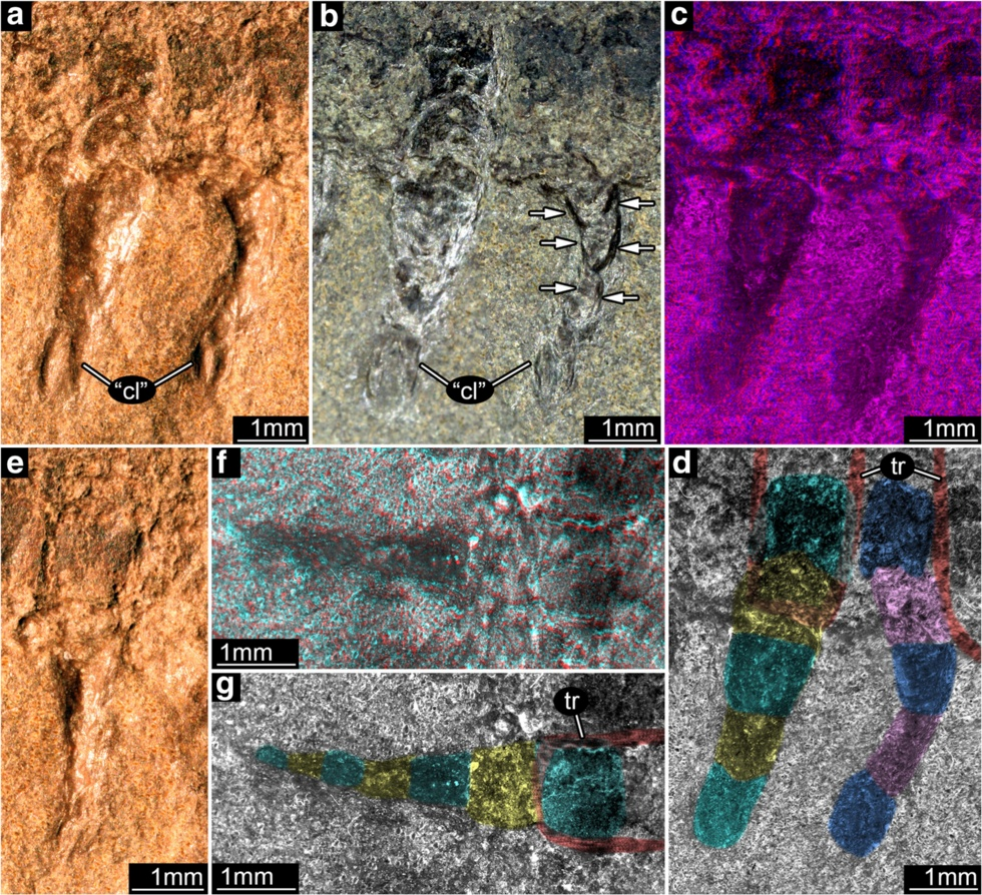
Thoracic and abdominal appendages of *Srokalarva berthei*. **a** to **d**, Second (mesothoracic) and third (metathoracic) appendages; (**e**) to (**g**), True first abdominal appendage. **a**, **e**, Directed low angle light. **b**, Undirected reflected light. Arrows mark patterns resembling supposed claw. **c**, Red-blue anaglyph of a virtual surface reconstruction. (Use red-cyan glasses to view.) **d**, Interpretative version of (**c**). **f**, Red-cyan stereo-anaglyph under cross-polarized light. (Use red-cyan glasses to view.) **g**, Interpretative version of (**f**). Abbreviations: “**cl**”, originally interpreted claw; **tr**, tergite rim

The thoracic segments do not differ markedly from the abdominal segments in the original interpretation. Our observations contradict this view. Three, well-delineated, nonoverlapping and noninterlinking regions of thoracic sclerites are apparent from an assessment of surface relief and color (Fig. 1a, d; Additional file 1: Figure S1A,D). The three thoracic legs are significantly more robust, longer and possess a greater diameter than the abdominal leglets. Originally thoracic legs were reconstructed with seven elements, whereas we found five major elements with a possible sixth element bearing terminal paired claws that are variably preserved (Fig. 3a–d). Although dark lines occur on sclerite surfaces and were interpreted originally as setae [6], and Mazon Creek fossils occasionally preserve fine hairs [15], we found no evidence for hirsute integument. Areas between the sclerites appear to preserve softer cuticle. These observations indicate that the thorax extends further rearward than the original reconstruction, corresponding to the anterior five postcephalic segments of Kukalová-Peck [6], and is more differentiated from the abdomen than originally reconstructed. The abdominal segments are in register (body segments matching respective leglets), but with overlapping tergites, the exact intersegmental boundaries are difficult to discern. This is borne out by a lack of an exact match of tergites between part and counterpart.

### The abdomen and leglets

**Fig. 4:**
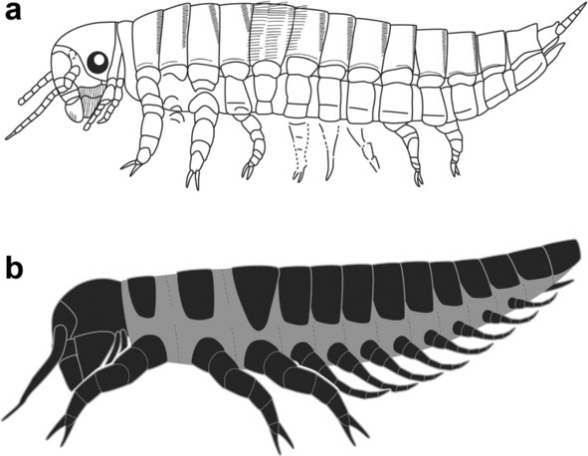
Reconstructions of *Srokalarva berthei*. **a**, The original interpretation, simplified from Kukalová-Peck (1991). **b**, A new reconstruction based on the present study. The grey hue indicates softer, more weakly sclerotized regions between sclerites. Dotted lines indicate possible intersegmental junctures which have been preserved

The original interpretation listed eleven abdominal segments (Fig. 4a) [6]. This number was arrived at by a miscount, with the two anteriormost “abdominal” segments [6] actually combined into our posteriormost metathoracic segment. Also, the initial count failed to recognize an inconspicuous but preserved segment behind the presumptive ‘cerci’. Consequently, ten abdominal segments are recognized in the current restoration (Fig. 4b). The first eight of these have ventral appendicular leglets (Figs. 1, 3e–g, Additional file 1: Figure S2). Originally, the last abdominal segment bore a pair of segmented ‘cerci’. This structure is apparent in relief and color, but its presumptive segmental subdivision likely is an artifact caused by irregularities of nonbiological surfaces. Due to the segmental mismatch in the original reconstruction, the supposed cerci do not arise from the eleventh, but from our ninth abdominal segment and are interpreted as ventrally positioned, paired, precursor genitalia ontogenetically comparable to urogomphi.

The abdominal appendages differ in certain aspects from the original interpretation. The abdominal appendages are inserted more dorsad along the abdominal sidewall, or pleurite, than formerly recognized, and are partially covered by the body. This indicates longer appendages than originally reconstructed, but they are somewhat shorter than the thoracic ones. None of these appendages preserve unequivocally a distal claw. As the exact subdivision of these appendages is difficult to discern, there appears to be two broader proximal elements and up to five smaller, distally tapering ones. The abdominal appendages therefore are composed of more elements and are more markedly gracile than the thoracic ones.

## Discussion

**Fig. 5:**
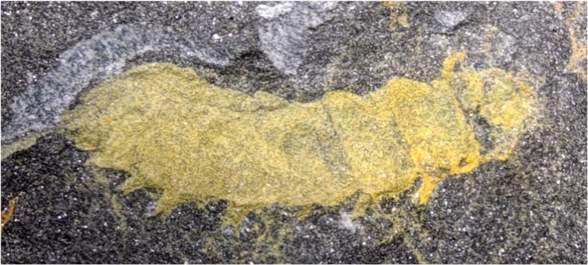
*Metabolarva bella*, from the Late Pennsylvanian of Germany [8]

**Fig. 6:**
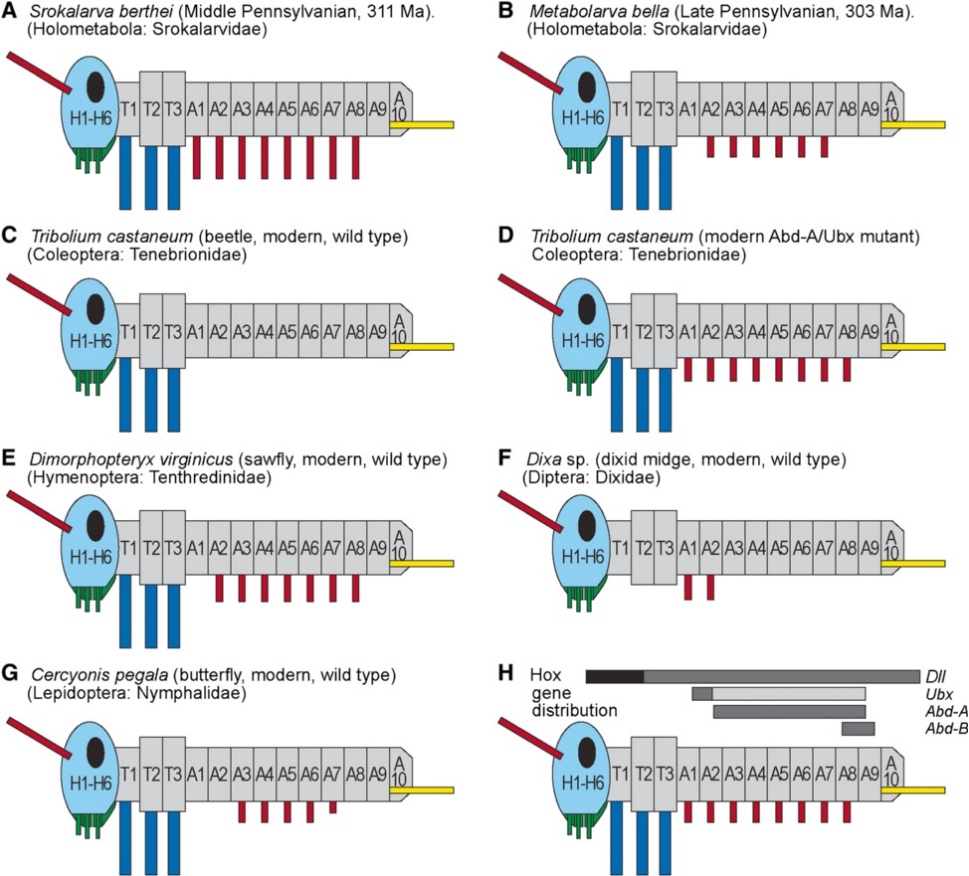
Expression domains of abdominal Hox genes in larvae of the earliest holometabolans, the beetle *Tribolium castaneum*, and other modern taxa. The expression of *Ubx*, *Abd-A* and *Abd-B* Hox genes in larvae of the earliest holometabolans are shown at (**a**, **b**). The most closely related model species affiliated with the earliest holometabolans probably is the beetle *Tribolium castaneum*, whose pattern of Hox gene development is shown in the wild-type at (**c**), and an *Ubx/Abd-A* mutant in (**d**). A sample of the Hox gene effects on abdominal leglet development are given in (**e**–**g**), showing the variety of expression patterns on abdominal appendages in taxa of the Hymenoptera, Diptera and Lepidoptera. The expression domains of *Distalless* (*Dll*), and the Hox genes *Ultrabithorax* (*Ubx*), *Abdominal-A* (*Abd-A*) and *Abdominal-B* (*Abd-B*) [15] in holometabolous larvae is shown at (**h**). The colors represent, from anterior to posterior: orange, antennae; green, mouthparts; blue, thoracic legs; red, abdominal leglets; and yellow, cerci. The stemmata likely are present and the cerci are inferred in *Srokalarva berthei*

The placement of serially homologous hexapod segments and their appendages is under the early developmental regulation by discrete clusters of homeotic genes, or Hox genes [16]. Several genes could have regulated the expression or nonexpression of abdominal appendages, such as leglets of *Srokalarva berthei* (Fig. 4) and *Metabolarva bella* (Fig. 5) [17]. The gene *Distalless* (*Dll*) is responsible for evaginations of the body wall that often result in development of segmental appendages such as certain mouthparts, antennae and walking legs [16]. This general function of *Dll*, in combination with Hox genes such as *abd-A*, are commissioned to express or repress development of appendages in certain body regions, such as the ventral abdominal leglet series of segments A1–A8 in many insects [15], unless it is repressed by the Hox genes *Ultrabithorax* (*Ubx*), *Abdominal-A* (*abd-A*) or their functional equivalents (Fig. 6h) [18]. Repression of *Dll* by *Ubx* or *abd-A* is well documented in a variety of model insects, particularly the holometabolous larvae of the red flour beetle *Tribolium castaneum* (Coleoptera) (Fig. 6c, d) [15, 19], pomace fly *Drosophila melanogaster* (Diptera) [20], silkworm *Bombyx mori* (Lepidoptera) [21], and other holometabolous insects (Fig. 6e–g). These genetic switches are also known for more basal insect lineages such as the cricket *Gryllus bimaculatus* [22] and grasshopper *Schistocerca gregaria* (Orthoptera) [23], and more remotely, the springtail *Orchesella cincta* (Collembola) [24], a non-insect hexapod. Although developmental capacity for expression and repression of abdominal leglets was inherited by the earliest holometabolous lineages [25], it appears that the oldest eruciform larvae had a conservative abdominal Hox-gene developmental pattern, with a complete series of leglets on segments A1–A8 in *Srokalarva berthei* (Figs. 4, 6a) and A2–A7 in *Metabolarva bella* (Figs. 5, 6b) the latter indicating that the *abd-A* gene repressed leglet expression in segment A1.

The presence of fully developed abdominal leglets in *Srokalarva berthei* (Fig. 6a), was followed ca. 8 million-years later by more diminutive leglets and the absence of the anteriormost (A1) and posteriormost (A8) leglets in the abdominal series of *Metabolarva bella* (Fig. 6b). This latter condition indicates that activation of the *Ubx*/*Abd-A*/*Abd-B* repressor genes may have occurred within basalmost holometabolans, even though these genes were latently present in their ancestors [22]. These relatively simple patterns likely were superseded by more complex regulatory networks involving *Abdominal-B* (*abd-B*) genes and complicated by a series of activator and repressor factors that bound with regulatory proteins to express or repress appendage development [18, 26]. Evidence for this includes extant basal lineages of Coleoptera and Hymenoptera, which have more complex abdominal leglet development and distribution patterns [27, 28] than those of *Srokalarva berthei* or *Metabolarva bella*.

A campodeiform body facies and life habits has been favored as the ancestral endopterygote larva, characterized by an active, predaceous, dorsoventrally flattened larva with forwardly-directed mouthparts. Recently, a hypothesis that the ancestral endopterygote larva was eruciform has gained broader acceptance, typified by a cylindrical, sluggish, herbivorous, caterpillar-like larva with downwardly-directed mouthparts. The morphology of *Srokalarva berthei* and *Metabolarva bella* [8] supports the eruciform hypothesis, although such an attribution may represent an oversimplification [29, 30]. The eight million-year-older *Srokalarva berthei* has features that hint at a campodeiform habitus, such as limited dorsoventral flattening (Additional file 1: Figure S3) and laterally extended legs (Fig. 4b). Most features, including a caterpillar form, presence of abdominal leglets typically used for anchoring to vegetation, and downwardly directed mouthparts featuring broadly attached, herbivore mandibles [31], indicate an eruciform larva. Plant damage from an unknown external feeder on contemporaneous medullosan foliage of *Macroneuropteris scheuchzeri* [32] would be expected from a *Srokalarva berthei* larva. Additionally, a legless eruciform larva similar to *Srokalarva berthei* could have been responsible for creating primitive, elongate pith galls in the frond rachises of marattialean *Psaronius* ferns a few million years later [33].

Within Holometabola, and based on the original description, *Srokalarva berthei* previously has been interpreted as an antliophoran [34]. Yet, the presence of supposed suture lines on the posterior head does not be support such an attribution. By contrast, the comparably long and well-developed abdominal appendages on eight segments do have a distinct comparison among extant larvae: the aquatic larvae of Megaloptera possess comparable appendages. Nevertheless, other aspects of the morphology of *Srokalarva berthei* are not compatible with a megalopteran identity. For example, the head morphology is rather unspecialized, while megalopterans have a prognathous head with long mandibles and rather short antennae. Still, the presence of eight abdominal appendages may be an autapomorphic specialization of *Srokalarva berthei* and not necessarily an ancestral feature of Holometabola. The presence of serial abdominal appendages could indicate that *Srokalarva berthei* is closely related to Megaloptera, but branched from this lineage [9, 10] and evolved a variety of features indicating specialized herbivory typical of a “primitive”, eruciform caterpillar [34], known from younger deposits [8].

## Conclusions

The unique specimen of *Srokalarva berthei* provides previous unknown data for understanding the early evolution of Holometabola, and an assessment of the various biological and environmental factors that could have affected the early evolution of this hyperdiverse group of insects. From a detailed evaluation of *Srokalarva berthei*, four general conclusions can be made.

1. Based on our morphological description of this specimen, the earliest holometabolous larvae were thick-bodied, cylindrical, inactive, herbivorous, and possessed a downwardly oriented head and mouthparts that are representative of eruciform larvae. This condition differs significantly from the alternative hypothesis of the earliest holometabolous larvae as thin-bodied, flattened, highly active, predatory forms with forwardly-directed head and mouthparts typical of campodeiform larvae.
2. The developmental biology of early holometabolous larvae of the Late Carboniferous soon involved homeotic gene regulation of abdominal leglets through repression of the *Distalless* gene by the *Abdominal-A* and *Ultrabithorax* genes. Activation of this regulatory mode may have happened during the Late Pennsylvanian, between the demise of *Srokalarva berthei* and the appearance of *Metabolarva bella*. This early experimentation in abdominal appendage developmental regulation generated the beginnings of abdominal appendage diversity seen today in the larvae of major holometabolous lineages.
3. Based on their morphologies, the diet of *Srokalarva berthei* and *Metabolarva bella* likely were herbivorous, as external foliage feeders. This inference is consistent with insect feeding damage occurring on a several seed-plant taxa in contemporaneous deposits.
4. The taxonomic affinities of *Srokalarva berthei* preferentially lies with the neuropteroid branch rather than the antliophoran branch of the Holometabola. This basal bifurcation of the Holometabola probably predates its separation into the major lineages of today.

## Methods

The single known specimen is preserved as part and counterpart and required special imaging methods for viewing and documentation. A broad habitus view of the entire specimen was provided by combining separate images into a composite form. Each component image was taken from separate focal planes that subsequently were integrated by CombineZM® from a series of images. These different images then were stitched with Adobe Photoshop® CS 3 software under cross-polarized light with a Canon Rebel T3i camera equipped with a MP-E 65 mm lens and a MT 24 EX Canon Twin Flash. For comparison of the specimen to earlier studies that used standard light-microscopy, fully reflected vertical light as well as directed side light from different angles was used. In order to document specimen relief, two techniques were applied. The first approach was stereo-imaging that employed the Canon Rebel T3i camera. A second procedure involved virtual surface reconstruction resulting from stacks of images recorded on a Zeiss Axiophot microscope attached to a Skopetech DCM 510 camera. Cross-polarized light was provided by multiple, optical-fiber light sources. Virtual surfaces were calculated in Image Analyzer® software.

### Ethics statement

Our study did not involve the use any human participants or animals.

## Additional file

Additional file 1:
**Text S1. Geological and paleobiological setting.** This section provides a summary of the depositional environment, type of general biological and geological setting, and other terrestrial and freshwater organisms inhabiting the Mazon Creek site in northeastern Illinois, ca. 311 million years ago, when *Srokalarva* was entombed. **Text S2. Taphonomic context of** ***Srokalarva berthei.*** This section provides details on the preservational style of Mazon Creek fossils in general, and the types of physical and chemical alteration of the sediments that gave rise to the types of features seen today on *Srokalarva berthei*. **Text S3. Systematics.** This section provides a formal description of *Srokalarva berthei* gen. et sp. nov., and its broader, inclusive, supra-generic monophyletic group. The subsections include: (i) the principal supra-generic characters; (ii) an extended synonymy; (iii) the material examined; (iv) the repository and collection accession number of the specimen; (v) a description of the holotype specimen that includes general remarks, the general habitus, and a detailed segment-by-segment description of the tagmatization and dorsal organization of the head, thorax and abdomen; and (vi) taxonomic identity of *Srokalarva berthei*. **Text S4. References.** This provides 20 reference citations to all text of the additional files. **Figure S1. Specimen part of MCP-322 imaged by variable illumination settings.** **Figure S2. Specimen counterpart of MCP-322 imaged by variable illumination settings. Figure S3. Interpretation of matrix embedment angle of** ***Srokalarva berthei.***
