# Erratum to: Life habits, hox genes, and affinities of a 311 million-year-old holometabolan larva

**DOI:** 10.1186/s12862-016-0725-x

**Published:** 2016-08-25

**Authors:** Joachim T. Haug, Conrad C. Labandeira, Jorge A. Santiago-Blay, Carolin Haug, Susan Brown

**Affiliations:** 1 Ludwig Maximilians University Munich, Biocenter – Department of Biology II and GeoBio-Center, Großhaderner Str. 2, Planegg-Martinsried, 82152 Germany; 2 Department of Paleobiology, National Museum of Natural History, Smithsonian Institution, Washington, DC 20013 USA; 3 Department of Entomology, University of Maryland, College Park, MD 20742 USA; 4 College of Life Sciences, Capital Normal University, Beijing, 100048 China; 5 Department of Crop and Agroenvironmental Sciences, University of Puerto Rico, Mayagüez, PR 00681 USA; 6 Division of Biology, Kansas State University, Manhattan, KS 66502 USA

## Erratum

Following the publication of this article [1] it has been brought to the attention of the authors and the editors of *BMC Evolutionary Biology* that the description of the newly described genus and species *Srokalarva berthei* did not fully meet the criteria of availability as defined by the ICZN (International Code of Zoological Nomenclature). The species description and accompanying ZooBank identification numbers were contained in an additional file and not in the main manuscript as required under the ICZN code.

This addendum is to ensure that the ICZN criteria for the availability of new names are satisfied. The following systematics section is identical to that published as part of additional file 1 of this article [1], but is republished here to ensure its full availability. The date of publication of the nomenclatural acts is the date that this addendum has been published.

### Systematics

Insecta, rank uncertain

Holometabola, rank uncertain

Srokalarvidae, tax. nov.

**Principle suprageneric characters.** Adults are unknown; the diagnostic features are based on larval characters, as detailed below.

### Extended synonymy.

1990 “oldest known larva” – Shear & Kukalová-Peck, p. 1827 [2].

1990 “oldest known endopterygote larva” – Shear & Kukalová-Peck, fig. 44 [2].

1991 “oldest known fossil larva” – Kukalová-Peck p. 151 [3].

1991 “oldest known endopterygote larva” – Kukalová-Peck p. 171; fig. 6.26 A [3].

1997 “first Carboniferous larva” – Kukalová-Peck, p. 204 [4]

1997 “oldest known larva” – Kukalová-Peck, p. 204 [4].

1997 “Berthe-Traub specimen” – Kukalová-Peck, p. 204 [4].

1997 “Berthe-Traub larva” – Kukalová-Peck, p. 204 [4].

1997 oldest true larva – Kukalová-Peck, p. 206 [4].

1997 “*Srokalarva berthei* “– Kukalová-Peck, fig. 14B.11 (first use of the Linnaean binomial) [4].

1997 “Alleged Carboniferous endopterygote larva” – Willmann, fig. 20.5 [5].

1997 “alleged Carboniferous larva” – Willmann, p. 276 [5].

1997 “specimen described as Carboniferous holometabolan larva“– Willmann, p. 276 [5].

1997 “alleged endopterygote Carboniferous larva” – Willmann, p. 277 [5].

1997 “oldest known endopterygote larva'”– Willmann, p. 277 [5].

2002 “alleged holometabolan larva from the Westphalian of Mazon Creek” – Rasnitsyn & Quicke, p. 157 [6].

2002 “*Srokalarva*”– Labandeira & Santiago-Blay, pp. 101-102 [7].

2005 “*Srokalarva*”– Grimaldi & Engel, p. 54 [8].

2007 “alleged holometabolous larva from the Late Carboniferous” – Nel et al. p. 350 [9].

2011 “*Srokalarva berthei*” – Labandeira, pp. 11, 16; tab 1 [10].

2011 “*Srokalarva*”– Labandeira, pp. 11, 335 [10].

2011 “*Srokalarva berthei*”– Labandeira, fig. 1G [10].

2013 *Srokalarva bertei* – Nel et al. p. 259, fig. 3; (sic!) [11].

2013 *Srokalarv*a – Nel et al., table 3 [11].

Genus *Srokalarva* gen. nov.

Species: *berthei* sp. nov.

### Material

The fossil consists of an ironstone concretion from the Mazon Creek fossil megalocality, collected at Pit 11 near Essex, in northwestern Kankakee, Co. Illinois, U.S.A. [12, 13]. *Srokalarva berthei* was found in the Francis Creek Shale Member of the Carbondale Formation. The age of the fossil is the regional Upper Desmoinesian Stage of eastern North American chronostratigraphy, corresponding to the Westphalian D interval of the older European geochronology [13], and is considered of late Moscovian Age, ca. 311 Ma, based on the most recent time scale [14].

### Holotype

Specimen MCP-322 is deposited in the Geology Department of the Field Museum of Natural History, Chicago, Illinois, USA. This specimen previously was housed in the collections of the Mazon Creek Project of Northeastern Illinois University, in Chicago, Illinois.

*Remarks―*The designation, *Srokalarva berthei*, was not validated by the fourth edition of the *International Code of Zoological Nomenclature* at the time that the Linnaean binomen was applied to this fossil in 1997 [4]. This lack of formal validation is attributed to (i) the absence of a valid description, (ii) a diagnosis was not offered, and (iii) a holotype specimen was not designated, thus rendering the name, *Srokalarva berthei*, a nomen nudum at first mention. Nevertheless, the *Srokalarva berthei* binomen is available and is used in this report. The formal species description of *Srokalarva berthei* Haug, Labandeira, Santiago-Blay, Haug and Brown is provided under the ZooBank identification number ADDB3CD0-68 F1-4591-93BB-1C56AD9F8E10. ZooBank is accessed through http://zoobank.org.

The description follows the scheme for arthropods proposed by Haug et al. [15]; whereby separate descriptions are provided for each segment. For each description, there is initial concentration on dorsal organization, followed by structural details for each appendage or other specialized structure. Terminology is kept at a more general level, yet specialized terms for different groups, especially involving insect and mandibulate terminology, are given. This approach should improve comparisons to other arthropod groups and a better understanding arthropod biology for the non-expert reader.

### General habitus

Elongate sclerotized arthropod of about 22 mm length. Body organized into 18 segments (17 externally visible, one inferred).

We have used the formal taxon, Holometabola, as an unranked, supraordinal designation that encompasses all holometabolous insects. Holometabolous insects are characterized by the unique feature of complete metamorphosis, featuring a dramatic change in ontogenetic development from one major life-stage to the next, and typified by the life stages of egg to larva to pupa to adult. In addition, the larval stage has a distinctive type of wing development, the endopterygote condition, which refers to the internal formation of wings under the thoracic cuticle. The term, endopterygote, thus is restricted to this type of wing development, although we recognize that historically the term, “Endopterygota”, has been used as a synonym for Holometabola [16].

### Tagmatization and dorsal body organization

**Head**. Anterior five segments, ocular segment and post-ocular segments 1-5 (post-ocular segment 2 inferred by comparison with modern forms), dorsally forming a continuous head capsule. All these segments are head segments. Length of head about 2.9 mm. A possible suture line is apparent either between postocular segments 1 and 2 or between 2 and 3.

**Thorax**. Post-ocular segments 6–8 subsimilar in dorsal morphology; they are recognized therefore as a separate tagma. (The *Srokalarva berthei* thorax is not necessarily directly homologous to the thorax in other arthropods; however, it is homologous to the pro-, meso- and metathoraces of other insects.) Postocular segment 6 (thorax segment 1, the prothorax) dorsally forming a sclerotized tergite (pronotum) of roughly rounded-rectangular in outline. Pronotum surrounded apparently by softer membrane, not directly articulating or covering posterior parts of the head or the tergite of the succeeding segment. Length of pronotum about 1.4 mm; length of membranous area anterior to tergite about 0.3 mm. Exact dorsalventral dimension difficult to assess due to a slightly tilted body embedded in matrix.

Postocular segment 7 (thorax segment 2, the mesothorax) dorsally forming a sclerotized tergite (mesonotum). Mesonotum surrounded apparently by softer membrane anteriorly, not directly articulating to tergite of the preceding or succeeding segment. Length of mesonotum about 1.3 mm; length of membranous area anterior to tergite about 0.9 mm. Exact dorsalventral dimension difficult to assess due to slightly tilted embedment, but larger in dorsalventral dimension than pronotum.

Postocular segment 8 (thorax segment 3, the metathorax) dorsally forming a sclerotized tergite (metanotum). Metanotum surrounded by apparently softer membrane, not directly articulating to tergite of the preceding segment. Length of mesonotum about 2.2 mm; length of membranous area anterior to tergite about 0.6 mm. Exact dorsalventral dimension difficult to assess due to slightly tilted embedding, but larger in dorsalventral dimension than mesonotum.

**Abdomen**. Postocular segments 9–18 subsimilar in dorsal morphology; they are recognized therefore as a separate tagma, the abdomen (not homologous to abdomen in other arthropods).

Postocular segment 9 (abdominal segment 1) dorsally (and “laterally”) forming a sclerotized tergite. Tergite in direct contact to preceding tergite (metanotum), slightly overhanging next posterior one. Length about 1.5 mm. Exact dorsalventral dimension difficult to assess due to slightly tilted embedment, but slightly shorter in dorsalventral dimension than metanotum.

Postocular segment 10 (abdominal segment 2) dorsally (and “laterally”) forming a sclerotized tergite. Tergite slightly overhanging adjacent posterior one. Length about 1.4 mm. Exact dorsalventral dimension difficult to assess due to slightly tilted embedment, but slightly shorter in dorsalventral dimension than preceding tergite.

Postocular segment 11 (abdominal segment 3) dorsally (and “laterally”) forming a sclerotized tergite. Tergite slightly overhanging adjacent posterior one. Length about 1.1 mm. Exact dorsalventral dimension difficult to assess due to slightly tilted embedment, but slightly shorter in dorsalventral dimension than preceding tergite.

Postocular segment 12 (abdominal segment 4) dorsally (and “laterally”) forming a sclerotized tergite. Tergite slightly overhanging adjacent posterior one. Length about 1.1 mm. Exact dorsalventral dimension difficult to assess due to slightly tilted embedment, but slightly shorter in dorsalventral dimension than preceding tergite.

Post-ocular segment 13 (abdominal segment 5) dorsally (and “laterally”) forming a sclerotized tergite. Tergite slightly overhanging adjacent posterior one. Length about 1.1 mm. Exact dorsalventral dimension difficult to assess due to slightly tilted embedment, but slightly shorter in dorsalventral dimension than preceding tergite.

Postocular segment 14 (abdominal segment 6) dorsally (and “laterally”) forming a sclerotized tergite. Tergite slightly overhanging adjacent posterior one. Length about 1.1 mm. Exact dorsalventral dimension difficult to assess due to slightly tilted embedment, but slightly shorter in dorsalventral dimension than preceding tergite.

Postocular segment 15 (abdominal segment 7) dorsally (and “laterally”) forming a sclerotized tergite. Tergite slightly overhanging adjacent posterior one. Length about 1.0 mm. Exact dorsalventral dimension difficult to assess due to slightly tilted embedment, but slightly shorter in dorsalventral dimension than preceding tergite.

Postocular segment 16 (abdominal segment 8) dorsally (and “laterally”) forming a sclerotized tergite. Tergite slightly overhanging adjacent posterior one. Length about 0.8 mm. Exact dorsalventral dimension difficult to assess due to slightly tilted embedment, but slightly shorter in dorsalventral dimension than preceding tergite.

Postocular segment 17 (abdominal segment 9) dorsally (and “laterally”) forming a sclerotized tergite. Tergite slightly overhanging adjacent posterior one. Length about 1.0 mm. Exact dorsalventral dimension difficult to assess due to slightly tilted embedment, but slightly shorter in dorsalventral dimension than preceding tergite.

Postocular segment 18 (abdominal segment 10) dorsally (and “laterally”) forming a sclerotized tergite. Tergite slightly overhanging adjacent posterior one. Length about 1.5 mm. Exact dorsalventral dimension difficult to assess due to slightly tilted embedment, but slightly shorter in dorsalventral dimension than preceding tergite.

It remains unclear whether a possible eleventh abdominal segment is truly absent or simply not preserved.

### Structural details within each tagma

**Head**. Ocular segment without traces of compound eyes. Stemmata may be present but cannot be verified. Clypeo-labral complex well developed anteriorly on the head capsule. Clypeus about 0.8 mm in proximal-distal axis; labrum about 1.5 mm in proximal-distal axis.

Postocular segment 1 with a pair of well-developed appendages, antennae (antennulae in other mandibulates) inserted dorsad to the clypeus. Diameter at base about 0.4 mm; tapering distally, and stronger in the terminal third. Maximum preserved length about 4.8 mm. Preserved position indicates an original subdivision into numerous elements, yet no clear subdivisions are apparent.

No details of postocular segment 2 (intercalary segment) available.

Postocular segment 3 with a pair (?) of well-developed appendages, mandibles. Consisting of a single element each. Only visible in lateral view. Elements massive in appearance and of triangular outline, base of the triangle proximally, tip distally. Base of the triangle about 1.8 mm long, height of triangle about 2.3 mm. Dorsad to mandibles small structure compressed through head capsule; square-shaped in lateral view, with about 0.5 mm along one edge, possible representing the hypopharynx (possibly homologous to paragnaths in other mandibulates).

Postocular segment 4 with a pair (?) of well-developed appendages, maxillae (maxillulae in other mandibulates). Preserved position indicates an original subdivision into numerous elements, with a proximal part and a distal part (palp), yet no clear subdivisions are apparent. Distal part about 0.25 mm in diameter; overall length (proximal-distal axis) about 2.6 mm.

Postocular segment 5 with a presumably fused pair (?) of well-developed appendages, labium (maxillae or second maxillae in other mandibulates). Preserved position indicates an original subdivision into numerous elements, with a proximal part and a distal part (palp), yet no clear subdivisions are apparent. Distal part about 0.25 mm in diameter; overall length (proximal-distal axis) about 3.4 mm.

**Thorax**. Postocular segment 6 (prothorax) with a pair (?) of well-developed appendages. General shape in lateral view elongate; tapering distally. Proximal-distal length about 5.5 mm. Apparently subdivided into five major elements (possibly corresponding to coxa, trochanter, femur, tibia and tarsus), all subsimilar in length, exact dimension difficult to measure, and a distal part (pretarsus?) bearing a pair of claws.

Postocular segment 7 (mesothorax) with a pair (?) of well-developed appendages. General shape in lateral view elongate; tapering distally. Proximal-distal length about 6.2 mm (exact length difficult to assess). Apparently subdivided into five major elements (possibly corresponding to coxa, trochanter, femur, tibia and tarsus), all subsimilar in length, exact dimension difficult to measure; a distal part (pretarsus?) with claws is not preserved, but was most likely present.

Postocular segment 8 (metathorax) with a pair of well-developed appendages. General shape in lateral view elongate; tapering distally. Proximal-distal length about 6.1 mm (exact length difficult to assess). Apparently subdivided into five major elements (possibly corresponding to coxa, trochanter, femur, tibia and tarsus), all subsimilar in length, exact dimension difficult to measure; a distal part (pretarsus?) with claws is not preserved, but was most likely present.

**Abdomen**. Postocular segment 9 (abdominal segment 1) with a pair of well-developed appendages. General shape in lateral view elongate; tapering distally, and stronger than thoracic appendages. Proximal-distal length about 5.0 mm (exact length difficult to assess). Apparently subdivided into 7 major elements, all subsimilar in length, exact dimension difficult to measure; a distal pair of claws could not be observed.

Postocular segment 10 (abdominal segment 2) with a pair (?) of well-developed appendages. General shape in lateral view elongate; tapering distally, and stronger than thoracic appendages. Proximal-distal length difficult to assess, as distal part appears to be preserved incompletely. Probably originally resembling appendage of preceding segment, i.e. subdivided into 7 major elements, all subsimilar in length; a distal pair of claws could not be observed.

Postocular segment 11 (abdominal segment 3) with a pair (?) of well-developed appendages. General shape in lateral view elongate; tapering distally, and stronger than thoracic appendages. Proximal-distal length difficult to assess, as distal part appears to be preserved incompletely. Probably originally resembling appendage of preceding segment, i.e. subdivided into 7 major elements, all subsimilar in length; a distal pair of claws could not be observed.

Postocular segment 12 (abdominal segment 4) with a pair (?) of well-developed appendages. General shape in lateral view elongate; tapering distally, and stronger than thoracic appendages. Proximal–distal length difficult to assess, as distal part appears to be preserved incompletely. Probably originally resembling appendage of preceding segment, i.e. subdivided into 7 major elements, all subsimilar in length; a distal pair of claws could not be observed.

Postocular segment 13 (abdominal segment 5) with a pair (?) of well-developed appendages. General shape in lateral view elongate; tapering distally, and stronger than thoracic appendages. Proximal–distal length difficult to assess, but apparently about the same length as abdominal appendage 1. Probably originally resembling appendage of preceding segment, i.e. subdivided into 7 major elements, all subsimilar in length; a distal pair of claws could not be observed.

Postocular segment 14 (abdominal segment 6) with a pair (?) of well-developed appendages. General shape in lateral view elongate; tapering distally, and stronger than thoracic appendages. Proximal–distal length difficult to assess, as distal part appears to be preserved incompletely. Probably originally resembling appendage of preceding segment, i.e. subdivided into 7 major elements, all subsimilar in length; a distal pair of claws could not be observed.

Postocular segment 15 (abdominal segment 7) with a pair (?) of well-developed appendages. General shape in lateral view elongate; tapering distally, and stronger than thoracic appendages. Proximal–distal length difficult to assess, only a rather proximal part is preserved. Probably originally resembling appendage of preceding segment, i.e. subdivided into 7 major elements, all subsimilar in length; a distal pair of claws could not be observed.

Postocular segment 16 (abdominal segment 8) possibly originally with a pair (?) of well-developed appendages, but no structure is preserved.

Postocular segment 17 (abdominal segment 9) with a pair of short, conical, backward pointing appendages of slightly less than 2 mm in length. Post-ocular segment 18 (abdominal segment 10) appears to lack appendages.

### Taxonomic Identity of *Srokalarva berthei*

Four possibilities exist for the possible identity of *Srokalarva berthei*. A first hypothesis is that *Srokalarva berthei* plausibly could be interpreted as an isopod or isopod-like crustacean. Division of *Srokalarva berthei* into three, distinctive tagma, a head, thorax and abdomen, would augur against such an affiliation. Additionally, the fivefold external organization of the head into discrete ocular, intercalary, mandibular, maxillary and labial regions, from anatomical anterior to posterior position, is a condition not seen in any crustacean.

Several authors [5, 8] have indicated that *Srokalarva berthei* represents a myriapod. This interpretation appears based on the assumption that myriapods have a homonomous trunk tagmosis, which was reconstructed originally for *Srokalarva berthei*. However, myriapods do not possess a homonomous trunk tagmosis [17]. More importantly, our reinvestigation demonstrates that the trunk of *Srokalarva berthei* is indeed differentiated into two tagmata, a thorax with three segments and an abdomen with ten (externally visible) segments. Such a tagmosis pattern clearly indicates an insectan affinity for *Srokalarva berthei*.

Could *Srokalarva berthei* represent an apterygote form instead of a larval insect? The mouthparts of *Srokalarva berthei* are clearly ectognathous, and an entognath affinity, typical of the Protura, Collembola and Diplura can be excluded. By virtue of its larval status, *Srokalarva berthei* lacks characters that would indicate an affinity to Zygentoma or Archaeognatha, or a position somewhere close to the node of Ectognatha or Pterygota. The general morphology, especially of the thorax, also does not indicate a nymphal identity, such as a hemimetabolous insect.

The general morphology of *Srokalarva berthei* is compatible with an interpretation as an endopterygote (holometabolous) larva. Propositions that have been used to exclude an endopterygote interpretation can be shown to be misinterpretations, likely caused by fossil specimen artifacts. Major examples from publications using such features as an argument for exclusion of *Srokalarva berthei* from Endopterygota include the following misinterpretations and sources:

1. There is no pronounced tagmosis into thorax and abdomen [8].
2. There is no sequence of leg-bearing and apodous segments [6].
3. There are no externally visible compound eyes or ocelli [5].
4. There are no claws on abdominal appendages [5].
5. There are no "segmented" cerci [5].

The interpretation of *Srokalarva berthei* as an endopterygote larva is plausible based on the alternatives to these five characters. Other features show that *Srokalarva berthei* is an endopterygote larva. The morphology and arrangement of the tergites demonstrate an endopterygote condition. Distinct sclerites intercalated between a larger softer, occasionally membranous, area can be seen in various endopterygote larvae, including an eruciform larva from the Early Permian of Uralian Russia [18]. Thoracic legs with few elements are indicative of the larval nature of *Srokalarva berthei.* We conclude that there is no character contradicting the interpretation of *Srokalarva berthei* as a holometabolous larva, whereas there are several characters positively and parsimoniously supporting this attribution. Given the number of details now known, *Srokalarva berthei* is currently the best candidate to represent a true holometabolous larva during the Middle Pennsylvanian Period. Details of *Metabolarva bella* also make it a plausible candidate for a holometabolous larva [10], but a detailed description of its morphology is desirable. *Srokalarva berthei* probably represents a late, probably ultimate, larval instar based on complete abdominal segmentation of ten evident segments, the presence of likely genital structures, and its overall size.
