# Supplementary material for: Life habits, hox genes, and affinities of a 311 million-year-old holometabolan larva
Source: BMC Evol Biol. 2015 Sep 29;15:208. doi: 10.1186/s12862-015-0428-8 (PMC4587847; doi:10.1186/s12862-015-0428-8)
Supplement: Additional file 1: — Text S1. Geological and paleobiological setting. This section provides a summary of the depositional environment, type of general biological and geological setting, and other terrestrial and freshwater organisms inhabiting the Mazon Creek site in northeastern Illinois, ca. 311 million years ago, when Srokalarva was entombed. Text S2. Taphonomic context of Srokalarva berthei. This section provides details on the preservational style of Mazon Creek fossils in general, and the types of physical and chemical alteration of the sediments that gave rise to the types of features seen today on Srokalarva berthei. Text S3. Systematics. This section provides a formal description of Srokalarva berthei gen. et sp. nov., and its broader, inclusive, supra-generic monophyletic group. The subsections include: (i) the principal supra-generic characters; (ii) an extended synonymy; (iii) the material examined; (iv) the repository and collection accession number of the specimen; (v) a description of the holotype specimen that includes general remarks, the general habitus, and a detailed segment-by-segment description of the tagmatization and dorsal organization of the head, thorax and abdomen; and (vi) taxonomic identity of Srokalarva berthei. Text S4. References. This provides 20 reference citations to all text of the additional files. Figure S1. Specimen part of MCP-322 imaged by variable illumination settings. Figure S2. Specimen counterpart of MCP-322 imaged by variable illumination settings. Figure S3. Interpretation of matrix embedment angle of Srokalarva berthei. [file 12862_2015_428_MOESM1_ESM.doc]

ADDITIONAL FILES FOR:

**Life Habits, Hox Genes, and Affinities of a**

**311 Million-Year-Old Holometabolan Larva**

Joachim T. Haug, Conrad C. Labandeira, Jorge Santiago-Blay,

Carolin Haug and Susan Brown

**Contents**

Geological and paleobiological setting…………………………………………………….. 1

Taphonomic context of *Srokalarva* *berthei*………………………………………………… 3

Systematics…………………………………………………………………………………. 5

Principal suprageneric characters……………………………………………………….... 5

Extended synonymy……………………………………………………………………… 5

Material…………………………………………………………………………………… 6

Holotype………………………………………………………………………………….. 6

General habitus…………………………………………………………………………… 8

Tagmatization and dorsal body organization……………………………………………... 8

Head……………………………………………………………………………………. 8

Thorax………………………………………………………………………………….. 8

Abdomen……………………………………………………………………………….. 9

Structural details within each tagma……………………………………………………. 10

Head…………………………………………………………………………………... 10

Thorax………………………………………………………………………………… 11

Abdomen……………………………………………………………………………… 12

Taxonomic identity of *Srokalarva* *berthei*………………………………………………… 13

Supplemental data references……………………………………………………………... 15

Figure 1……………………………………………………………………………………. 18

Figure 2……………………………………………………………………………………. 19

Figure 3……………………………………………………………………………………. 20

**Additional file S1: Supplemental text. Geological and Paleobiological Setting**

Mazon Creek deposits containing the ironstone concretions from which *Srokalarva* *berthei* originates are part of the Francis Creek Shale that was formed ca. 311 million years ago during the Pennsylvanian (Late Carboniferous) Period. These deposits are interpreted as representing environments typical of a large deltaic system that was deposited by a major river that flowed toward the southwest, draining sediments from the Appalachian Orogeny to the northeast. The paleogeographic location of the Mazon Creek delta and associated deposits was within 10°N of the paleoequator, occurring under a subtropical environment adjacent coal-swamps, fluviatile environments, and a shallow marine delta and associated estuaries [S1]. Fossil carcasses, including plants and insects, were rapidly carried downstream and quickly buried while immediately incorporated in a layer of iron carbonate. Immediate sealing from an oxygenic environment, rapid lithification, and entombment with multiple events of mineralization resulted in the distinctive style of fossil preservation at Mazon Creek that is recognized today as a *Lagerstätte* of exceptional preservation and elevated diversity.

Mazon Creek is a megalocality, occurring as scattered spoil heaps from a regional network of open-pit mines that occupy four counties in northeastern Illinois, U.S.A. The coal seam from which the nodules originate are from the Middle Pennsylvanian Subperiod, and are noted for deposits consisting of fossil-bearing ironstone deposits that are geographically and environmentally subdivided into the shallow marine Essex Biota, and the freshwater and terrestrial Braidwood Biota [S1, S2]. The Essex Biota contains principally ctenophores; cnidarians; holothurian echinoderms; cephalopod, bivalve and gastropod mollusks; polychaete annelids; a diverse series of arthropods, mostly malacostracan crustaceans and eurypterids; a variety of cartilaginous and bony fish; and enigmatica such as *Tullimonstrum gregarium* [S2]. The Braidwood Biota is comprised principally of lycopsids, sphenopsids, pteridopsids and diverse assemblage of seed plants, including medullosans and cordaites; arthropods such as ostracods, freshwater and probably terrestrial horseshoe crabs; arachnids, millipedes, centipedes, isopods, freshwater shrimp and a diverse assemblage of insects that includes *Srokalarva* *berthei*; freshwater fish; and a modest variety of amphibian- and reptile grade tetrapods [S2]. The insect fauna is particularly diverse, and contains typical Palaeozoic lineages such as Diplura (telsontails); Monura (bristletails); Odonatoptera, consisting of Palaeozoic distant relatives of modern dragonflies; Ephemeroptera (mayflies); Paleodictyopteroidea, a diverse assemblage of superficially dragonfly-like insects with piercing-and-sucking mouthparts; Plecoptera (stoneflies); Dictyoptera, cockroach-like forms with long ovipositors; Orthoptera and related, poorly understood groups with a basal, grasshopper-like facies currently being studied; the enigmatic Miomoptera, probable precursors of Hemiptera; and Holometabola [S2, S3]. All sublineages of these insect groups at middle and lower taxonomic ranks are extinct.

**Additional file S2: Supplemental text. Taphonomic context of *Srokalarva* *berthei***

Our morphological interpretation of the specimen differs from that of Kukalová-Peck [S3‒S5]. These differences most likely result from the different techniques of documentation [S6]. Our alternative mode of documentation arises from the preservational style of certain fossils, such as those from Mazon Creek, which display two varied types of information regarding fossil structure [S7]. The first type of information involves changes in color and often involves a contrast in brightness between the biological structures of the fossil versus the nonbiological surface of the matrix. For Mazon Creek ironstone nodules, certain structures are outlined in black and yellowish hues. In addition, blue- and green hued structures can be recognized against reddish to brown or ochre colored matrix. A second type of information consists of surface relief, or texture, essential for identifying overlying structures. Such surface detail is important for distinguishing structures of the fossil from those of the matrix. Yet, relief can be difficult to understand, particularly when fossils are preserved in a three-dimensional mode with minimal compression. An additional complication is the trajectory plane of the split nodule, in which certain structures are in positive relief on one side but in negative relief on the opposite side. This phenomenon results in cleaved surfaces that are not mirror images, and the dividing split almost invariably is imperfect.

The standard way of documenting and investigating Mazon Creek fossils is under low-angle light which confounds these two types of information. Application of cross-polarized light enhances the color contrast and suppresses internally generated reflected light, or birefringence, sufficiently to separate information of color and brightness from that of surface relief and texture. Three-dimensional imaging techniques, such as stereo imaging or virtual surface reconstruction, under shadow-free light, can provide reliable data on surface relief and texture.

Kukalová-Peck [S4, S5] interpreted the specimen as preserved in a strictly lateral position. In her reconstruction, tergites and sternites are apparent. Our observation of the relief on the specimen (Suppl. Fig. 1) indicates that it is not preserved in strict lateral position, with the possible exception of the head (Suppl. Fig. 2). The thorax and abdomen of the specimen is preserved in laterodorsal aspect. The legs and especially their well-sclerotized insertions have become compressed through the tergites, and consequently are visible by virtue of their relief (Suppl. Fig. 3). The insertions also are recognizable by their dark, almost black color (Suppl. Figs 1, 2). These insertion regions appear originally to have been interpreted as the area where tergites and sternites meet at the lateral body wall. Nevertheless, from tracing the boundaries of the tergites, especially those of the thorax, it is clear that they are continuous along the entire preserved part of the body from dorsal to ventral aspect (Fig. 3). Consequently, we lack information regarding the structure of the ventral sternites. Additionally, in two cases, there are appendages of the opposite sides of the body that appear to have been preserved (Suppl. Fig. 1): the mesothoracic appendage and the first abdominal appendage. The length of these appendages in relation to the other leg/leglet series supports our observation that the specimen is deployed in a sagittally tilted position.

This interpretation has bearing on the presumed body shape of *Srokalarva* *berthei*. The specimen generally is interpreted as eruciform [S4, S5, S8, S9]; its body cross section was assumed to have been more-or-less circular. However, with an approximately circular cross section the appendage insertions could not have been dorsolaterally covered by tergites (Additional File. Fig. 3a, b). Only with dorsalventral or almost dorsalventral flattening of the body and tilting of the specimen onto the matrix, which is different than the head orientation, would there be covering of the appendage insertion bases by the tergites. Even if one assumes that the ventral tergites extended considerably further ventrally, along the body sidewall, a circular body cross section would not explain the observed preservation (Suppl. Fig. 3c, d). A covering of the appendage insertion areas by the tergites in dorsolateral embedment can only occur only if the body cross section was more oval, requiring slight dorsoventral flattening (Suppl. Fig. 3e, f). Thus, a somewhat flattened cross section likely was the original body morphology. A more complex model would suggest that the body had a circular cross section, was compressed dorsoventrally, then tilted and then finally embedded within the surrounding matrix. Although our proposal involves a hypothesized scenario with several assumptions, it remains the most parsimonious explanation that is consistent with a reasonable body structure and its subsequent movement prior to entombment within the matrix. We suggest that many of the original interpretations deviating from our observations are likely optical artifacts, a combination of internal birefringence issues and a mixing of information based differentiating shadow and intrinsic color. New imaging techniques allow for better retrieval of information preserved in exceptionally preserved fossil specimens [S10].

**Additional file S3: Systematics**

Insecta, rank uncertain

Holometabola, rank uncertain

Srokalarvidae, tax. nov.

**Principle suprageneric characters.** Adults are unknown; the diagnostic features are based on larval characters, as detailed below.

**Extended synonymy.**

1990 “oldest known larva” – Shear & Kukalová-Peck, p. 1827 [S4].

1990 “oldest known endopterygote larva” – Shear & Kukalová-Peck, fig. 44 [S4].

1991 “oldest known fossil larva” – Kukalová-Peck p. 151 [S5].

1991 “oldest known endopterygote larva” – Kukalová-Peck p. 171; fig. 6.26 A [S5].

1997 “first Carboniferous larva” – Kukalová-Peck, p. 204 [S3].

1997 “oldest known larva” – Kukalová-Peck, p. 204 [S3].

1997 “Berthe-Traub specimen” – Kukalová-Peck, p. 204 [S3].

1997 “Berthe-Traub larva” – Kukalová-Peck, p. 204 [S3].

1997 oldest true larva – Kukalová-Peck, p. 206 [S3].

1997 “*Srokalarva berthei* “– Kukalová-Peck, fig. 14B.11 (first use of the Linnaean binomial) [S3].

1997 “Alleged Carboniferous endopterygote larva” – Willmann, fig. 20.5 [S11].

1997 “alleged Carboniferous larva” – Willmann, p. 276 [S11].

1997 “specimen described as Carboniferous holometabolan larva“– Willmann, p. 276 [S11].

1997 “alleged endopterygote Carboniferous larva” – Willmann, p. 277 [S11].

1997 “oldest known endopterygote larva'”– Willmann, p. 277 [S11].

2002 “alleged holometabolan larva from the Westphalian of Mazon Creek” – Rasnitsyn & Quicke, p. 157 [S12].

2002 “*Srokalarva*”– Labandeira & Santiago-Blay, pp. 101-102 [S8].

2005 “*Srokalarva*”– Grimaldi & Engel, p. 54 [S13].

2007 “alleged holometabolous larva from the Late Carboniferous” – Nel et al. p. 350 [S14].

2011 “*Srokalarva berthei*” – Labandeira, pp. 11, 16; tab 1 [S9].

2011 “*Srokalarva*”– Labandeira, pp. 11, 335 [S9].

2011 “*Srokalarva berthei*”– Labandeira, fig. 1G [S9].

2013 *Srokalarva bertei* – Nel et al. p. 259, fig. 3; (sic!) [S15].

2013 *Srokalarv*a – Nel et al., table 3 [S15].

Genus *Srokalarva* gen. nov.

Species: *berthei* sp. nov.

**Material**

The fossil consists of an ironstone concretion from the Mazon Creek fossil megalocality, collected at Pit 11 near Essex, in northwestern Kankakee, Co. Illinois, U.S.A. [S1, S2]. *Srokalarva berthei* was found in the Francis Creek Shale Member of the Carbondale Formation. The age of the fossil is the regional Upper Desmoinesian Stage of eastern North American chronostratigraphy, corresponding to the Westphalian D interval of the older European geochronology [S2], and is considered of late Moscovian Age, ca. 311 Ma, based on the most recent time scale [S16].

**Holotype**

Specimen MCP-322 is reposited in the Geology Department of the Field Museum of Natural History, Chicago, Illinois, USA. This specimen previously was housed formerly in the collections of the Mazon Creek Project of Northeastern Illinois University, in Chicago, Illinois.

*Remarks―*The designation, *Srokalarva* *berthei*, was not validated by the fourth edition of the *International Code of Zoological Nomenclature* at the time that the Linnaean binomen was applied to this fossil in 1997 [S3]. This lack of formal validation is attributed to (i) the absence of a valid description, (ii) a diagnosis was not offered, and (iii) a holotype specimen was not designated, thus rendering the name, *Srokalarva* *berthei*, a nomen nudum at first mention. Nevertheless, the *Srokalarva* *berthei* binomen is available and is used in this report. The formal species description of *Srokalarva* *berthei* Haug, Labandeira, Santiago-Blay, Haug and Brown is provided under the ZooBank identification number ADDB3CD0-68F1-4591-93BB-1C56AD9F8E10. ZooBank is accessed through [http://zoobank.org](http://zoobank.org/).

The description follows the scheme for arthropods proposed by Haug et al. [S17]; whereby separate descriptions are provided for each segment. For each description, there is initial concentration on dorsal organization, followed by structural details for each appendage or other specialized structure. Terminology is kept at a more general level, yet specialized terms for different groups, especially involving insect and mandibulate terminology, are given. This approach should improve comparisons to other arthropod groups and a better understanding arthropod biology for the non-expert reader.

**General habitus**

Elongate sclerotized arthropod of about 22 mm length. Body organized into 18 segments (17 externally visible, one inferred).

We have used the formal taxon, Holometabola, as an unranked, supraordinal designation that encompasses all holometabolous insects. Holometabolous insects are characterized by the unique feature of complete metamorphosis, featuring a dramatic change in ontogenetic development from one major life-stage to the next, and typified by the life stages of egg to larva to pupa to adult. In addition, the larval stage has a distinctive type of wing development, the endopterygote condition, which refers to the internal formation of wings under the thoracic cuticle. The term, endopterygote, thus is restricted to this type of wing development, although we recognize that historically the term, “Endopterygota”, has been used as a synonym for Holometabola [S18].

**Tagmatization and dorsal body organization**

**Head**. Anterior five segments, ocular segment and post-ocular segments 1-5 (post-ocular segment 2 inferred by comparison with modern forms), dorsally forming a continuous head capsule. All these segments are head segments. Length of head about 2.9 mm. A possible suture line is apparent either between postocular segments 1 and 2 or between 2 and 3.

**Thorax**. Post-ocular segments 6‒8 subsimilar in dorsal morphology; they are recognized therefore as a separate tagma. (The *Srokalarva* *berthei* thorax is not necessarily directly homologous to the thorax in other arthropods; however, it is homologous to the pro-, meso- and metathoraces of other insects.) Postocular segment 6 (thorax segment 1, the prothorax) dorsally forming a sclerotized tergite (pronotum) of roughly rounded-rectangular in outline. Pronotum surrounded apparently by softer membrane, not directly articulating or covering posterior parts of the head or the tergite of the succeeding segment. Length of pronotum about 1.4 mm; length of membranous area anterior to tergite about 0.3 mm. Exact dorsalventral dimension difficult to assess due to a slightly tilted body embedded in matrix.

Postocular segment 7 (thorax segment 2, the mesothorax) dorsally forming a sclerotized tergite (mesonotum). Mesonotum surrounded apparently by softer membrane anteriorly, not directly articulating to tergite of the preceding or succeeding segment. Length of mesonotum about 1.3 mm; length of membranous area anterior to tergite about 0.9 mm. Exact dorsalventral dimension difficult to assess due to slightly tilted embedment, but larger in dorsalventral dimension than pronotum.

Postocular segment 8 (thorax segment 3, the metathorax) dorsally forming a sclerotized tergite (metanotum). Metanotum surrounded by apparently softer membrane, not directly articulating to tergite of the preceding segment. Length of mesonotum about 2.2 mm; length of membranous area anterior to tergite about 0.6 mm. Exact dorsalventral dimension difficult to assess due to slightly tilted embedding, but larger in dorsalventral dimension than mesonotum.

**Abdomen**. Postocular segments 9‒18 subsimilar in dorsal morphology; they are recognized therefore as a separate tagma, the abdomen (not homologous to abdomen in other arthropods).

Postocular segment 9 (abdominal segment 1) dorsally (and “laterally”) forming a sclerotized tergite. Tergite in direct contact to preceding tergite (metanotum), slightly overhanging next posterior one. Length about 1.5 mm. Exact dorsalventral dimension difficult to assess due to slightly tilted embedment, but slightly shorter in dorsalventral dimension than metanotum.

Postocular segment 10 (abdominal segment 2) dorsally (and “laterally”) forming a sclerotized tergite. Tergite slightly overhanging adjacent posterior one. Length about 1.4 mm. Exact dorsalventral dimension difficult to assess due to slightly tilted embedment, but slightly shorter in dorsalventral dimension than preceding tergite.

Postocular segment 11 (abdominal segment 3) dorsally (and “laterally”) forming a sclerotized tergite. Tergite slightly overhanging adjacent posterior one. Length about 1.1 mm. Exact dorsalventral dimension difficult to assess due to slightly tilted embedment, but slightly shorter in dorsalventral dimension than preceding tergite.

Postocular segment 12 (abdominal segment 4) dorsally (and “laterally”) forming a sclerotized tergite. Tergite slightly overhanging adjacent posterior one. Length about 1.1 mm. Exact dorsalventral dimension difficult to assess due to slightly tilted embedment, but slightly shorter in dorsalventral dimension than preceding tergite.

Post-ocular segment 13 (abdominal segment 5) dorsally (and “laterally”) forming a sclerotized tergite. Tergite slightly overhanging adjacent posterior one. Length about 1.1 mm. Exact dorsalventral dimension difficult to assess due to slightly tilted embedment, but slightly shorter in dorsalventral dimension than preceding tergite.

Postocular segment 14 (abdominal segment 6) dorsally (and “laterally”) forming a sclerotized tergite. Tergite slightly overhanging adjacent posterior one. Length about 1.1 mm. Exact dorsalventral dimension difficult to assess due to slightly tilted embedment, but slightly shorter in dorsalventral dimension than preceding tergite.

Postocular segment 15 (abdominal segment 7) dorsally (and “laterally”) forming a sclerotized tergite. Tergite slightly overhanging adjacent posterior one. Length about 1.0 mm. Exact dorsalventral dimension difficult to assess due to slightly tilted embedment, but slightly shorter in dorsalventral dimension than preceding tergite.

Postocular segment 16 (abdominal segment 8) dorsally (and “laterally”) forming a sclerotized tergite. Tergite slightly overhanging adjacent posterior one. Length about 0.8 mm. Exact dorsalventral dimension difficult to assess due to slightly tilted embedment, but slightly shorter in dorsalventral dimension than preceding tergite.

Postocular segment 17 (abdominal segment 9) dorsally (and “laterally”) forming a sclerotized tergite. Tergite slightly overhanging adjacent posterior one. Length about 1.0 mm. Exact dorsalventral dimension difficult to assess due to slightly tilted embedment, but slightly shorter in dorsalventral dimension than preceding tergite.

Postocular segment 18 (abdominal segment 10) dorsally (and “laterally”) forming a sclerotized tergite. Tergite slightly overhanging adjacent posterior one. Length about 1.5 mm. Exact dorsalventral dimension difficult to assess due to slightly tilted embedment, but slightly shorter in dorsalventral dimension than preceding tergite.

It remains unclear whether a possible eleventh abdominal segment is truly absent or simply not preserved.

**Structural details within each tagma**

**Head**. Ocular segment without traces of compound eyes. Stemmata may be present but cannot be verified. Clypeo-labral complex well developed anteriorly on the head capsule. Clypeus about 0.8 mm in proximal-distal axis; labrum about 1.5 mm in proximal-distal axis.

Postocular segment 1 with a pair of well-developed appendages, antennae (antennulae in other mandibulates) inserted dorsad to the clypeus. Diameter at base about 0.4 mm; tapering distally, and stronger in the terminal third. Maximum preserved length about 4.8 mm. Preserved position indicates an original subdivision into numerous elements, yet no clear subdivisions are apparent.

No details of postocular segment 2 (intercalary segment) available.

Postocular segment 3 with a pair (?) of well-developed appendages, mandibles. Consisting of a single element each. Only visible in lateral view. Elements massive in appearance and of triangular outline, base of the triangle proximally, tip distally. Base of the triangle about 1.8 mm long, height of triangle about 2.3 mm. Dorsad to mandibles small structure compressed through head capsule; square-shaped in lateral view, with about 0.5 mm along one edge, possible representing the hypopharynx (possibly homologous to paragnaths in other mandibulates).

Postocular segment 4 with a pair (?) of well-developed appendages, maxillae (maxillulae in other mandibulates). Preserved position indicates an original subdivision into numerous elements, with a proximal part and a distal part (palp), yet no clear subdivisions are apparent. Distal part about 0.25 mm in diameter; overall length (proximal-distal axis) about 2.6 mm.

Postocular segment 5 with a presumably fused pair (?) of well-developed appendages, labium (maxillae or second maxillae in other mandibulates). Preserved position indicates an original subdivision into numerous elements, with a proximal part and a distal part (palp), yet no clear subdivisions are apparent. Distal part about 0.25 mm in diameter; overall length (proximal-distal axis) about 3.4 mm.

**Thorax**. Postocular segment 6 (prothorax) with a pair (?) of well-developed appendages. General shape in lateral view elongate; tapering distally. Proximal-distal length about 5.5 mm. Apparently subdivided into five major elements (possibly corresponding to coxa, trochanter, femur, tibia and tarsus), all subsimilar in length, exact dimension difficult to measure, and a distal part (pretarsus?) bearing a pair of claws.

Postocular segment 7 (mesothorax) with a pair (?) of well-developed appendages. General shape in lateral view elongate; tapering distally. Proximal-distal length about 6.2 mm (exact length difficult to assess). Apparently subdivided into five major elements (possibly corresponding to coxa, trochanter, femur, tibia and tarsus), all subsimilar in length, exact dimension difficult to measure; a distal part (pretarsus?) with claws is not preserved, but was most likely present.

Postocular segment 8 (metathorax) with a pair of well-developed appendages. General shape in lateral view elongate; tapering distally. Proximal-distal length about 6.1 mm (exact length difficult to assess). Apparently subdivided into five major elements (possibly corresponding to coxa, trochanter, femur, tibia and tarsus), all subsimilar in length, exact dimension difficult to measure; a distal part (pretarsus?) with claws is not preserved, but was most likely present.

**Abdomen**. Postocular segment 9 (abdominal segment 1) with a pair of well-developed appendages. General shape in lateral view elongate; tapering distally, and stronger than thoracic appendages. Proximal-distal length about 5.0 mm (exact length difficult to assess). Apparently subdivided into 7 major elements, all subsimilar in length, exact dimension difficult to measure; a distal pair of claws could not be observed.

Postocular segment 10 (abdominal segment 2) with a pair (?) of well-developed appendages. General shape in lateral view elongate; tapering distally, and stronger than thoracic appendages. Proximal-distal length difficult to assess, as distal part appears to be preserved incompletely. Probably originally resembling appendage of preceding segment, i.e. subdivided into 7 major elements, all subsimilar in length; a distal pair of claws could not be observed.

Postocular segment 11 (abdominal segment 3) with a pair (?) of well-developed appendages. General shape in lateral view elongate; tapering distally, and stronger than thoracic appendages. Proximal-distal length difficult to assess, as distal part appears to be preserved incompletely. Probably originally resembling appendage of preceding segment, i.e. subdivided into 7 major elements, all subsimilar in length; a distal pair of claws could not be observed.

Postocular segment 12 (abdominal segment 4) with a pair (?) of well-developed appendages. General shape in lateral view elongate; tapering distally, and stronger than thoracic appendages. Proximal‒distal length difficult to assess, as distal part appears to be preserved incompletely. Probably originally resembling appendage of preceding segment, i.e. subdivided into 7 major elements, all subsimilar in length; a distal pair of claws could not be observed.

Postocular segment 13 (abdominal segment 5) with a pair (?) of well-developed appendages. General shape in lateral view elongate; tapering distally, and stronger than thoracic appendages. Proximal‒distal length difficult to assess, but apparently about the same length as abdominal appendage 1. Probably originally resembling appendage of preceding segment, i.e. subdivided into 7 major elements, all subsimilar in length; a distal pair of claws could not be observed.

Postocular segment 14 (abdominal segment 6) with a pair (?) of well-developed appendages. General shape in lateral view elongate; tapering distally, and stronger than thoracic appendages. Proximal‒distal length difficult to assess, as distal part appears to be preserved incompletely. Probably originally resembling appendage of preceding segment, i.e. subdivided into 7 major elements, all subsimilar in length; a distal pair of claws could not be observed.

Postocular segment 15 (abdominal segment 7) with a pair (?) of well-developed appendages. General shape in lateral view elongate; tapering distally, and stronger than thoracic appendages. Proximal‒distal length difficult to assess, only a rather proximal part is preserved. Probably originally resembling appendage of preceding segment, i.e. subdivided into 7 major elements, all subsimilar in length; a distal pair of claws could not be observed.

Postocular segment 16 (abdominal segment 8) possibly originally with a pair (?) of well-developed appendages, but no structure is preserved.

Postocular segment 17 (abdominal segment 9) with a pair of short, conical, backward pointing appendages of slightly less than 2 mm in length. Post-ocular segment 18 (abdominal segment 10) appears to lack appendages.

**Taxonomic Identity of *Srokalarva* *berthei***

Four possibilities exist for the possible identity of *Srokalarva berthei*. A first hypothesis is that *Srokalarva berthei* plausibly could be interpreted as an isopod or isopod-like crustacean. Division of *Srokalarva berthei* into three, distinctive tagma, a head, thorax and abdomen, would augur against such an affiliation. Additionally, the fivefold external organization of the head into discrete ocular, intercalary, mandibular, maxillary and labial regions, from anatomical anterior to posterior position, is a condition not seen in any crustacean.

Several authors [S11, S13] have indicated that *Srokalarva* *berthei* represents a myriapod. This interpretation appears based on the assumption that myriapods have a homonomous trunk tagmosis, which was reconstructed originally for *Srokalarva* *berthei*. However, myriapods do not possess a homonomous trunk tagmosis [S19]. More importantly, our reinvestigation demonstrates that the trunk of *Srokalarva* *berthei* is indeed differentiated into two tagmata, a thorax with three segments and an abdomen with ten (externally visible) segments. Such a tagmosis pattern clearly indicates an insectan affinity for *Srokalarva* *berthei*.

Could *Srokalarva* *berthei* represent an apterygote form instead of a larval insect? The mouthparts of *Srokalarva* *berthei* are clearly ectognathous, and an entognath affinity, typical of the Protura, Collembola and Diplura can be excluded. By virtue of its larval status, *Srokalarva berthei* lacks characters that would indicate an affinity to Zygentoma or Archaeognatha, or a position somewhere close to the node of Ectognatha or Pterygota. The general morphology, especially of the thorax, also does not indicate a nymphal identity, such as a hemimetabolous insect.

The general morphology of *Srokalarva berthei* is compatible with an interpretation as an endopterygote (holometabolous) larva. Propositions that have been used to exclude an endopterygote interpretation can be shown to be misinterpretations, likely caused by fossil specimen artifacts. Major examples from publications using such features as an argument for exclusion of *Srokalarva* *berthei* from Endopterygota include the following misinterpretations and sources:

1) There is no pronounced tagmosis into thorax and abdomen [S13].

2) There is no sequence of leg-bearing and apodous segments [S12].

3) There are no externally visible compound eyes or ocelli [S11].

4) There are no claws on abdominal appendages [S11].

5) There are no "segmented" cerci [S11].

The interpretation of *Srokalarva berthei* as an endopterygote larva is plausible based on the alternatives to these five characters. Other features show that *Srokalarva berthei* is an endopterygote larva. The morphology and arrangement of the tergites demonstrate an endopterygote condition. Distinct sclerites intercalated between a larger softer, occasionally membranous, area can be seen in various endopterygote larvae, including an eruciform larva from the Early Permian of Uralian Russia [S20]. Thoracic legs with few elements are indicative of the larval nature of *Srokalarva* *berthei.* We conclude that there is no character contradicting the interpretation of *Srokalarva berthei* as a holometabolous larva, whereas there are several characters positively and parsimoniously supporting this attribution. Given the number of details now known, *Srokalarva berthei* is currently the best candidate to represent a true holometabolous larva during the Middle Pennsylvanian Period. Details of *Metabolarva bella* also make it a plausible candidate for a holometabolous larva [S15], but a detailed description of its morphology is desirable. *Srokalarva berthei* probably represents a late, probably ultimate, larval instar based on complete abdominal segmentation of ten evident segments, the presence of likely genital structures, and its overall size.

**Additional file S4. Supplemental Data References**

S1. Selden PA, Nudds JR: *Evolution of Fossil Ecosystems.* Chicago: University of Chicago Press; 2005.

S2. Shabica C, Hay A, Editors: *Richardson’s Guide to the Fossil Fauna of Mazon Creek.* Chicago: Northern Illinois University Press; 1997.

S3. Kukalová-Peck J: **Mazon Creek insect fossils: the origin of insect wings and clues about the origin of insect metamorphosis.** In *Richardson’s Guide to the Fossil Fauna of Mazon Creek.* Edited by Shabica, CW Shabica, Hay AA. Chicago: Northern Illinois University Press; 1997:194‒207.

S4. Shear WA, Kukalová-Peck J: **The ecology of Paleozoic terrestrial arthropods: the fossil evidence.** *Can J Zool* 1990, **68**:1807‒1834.

S5. Kukalová-Peck J: **Fossil history and the evolution of hexapod structures.** In *Insects of Australia: A Textbook for Students and Research Workers.* 2^nd^ ed. Edited by Naumann ID, Carne PB, Lawrence JF, Nielsen ES, Spradbery JP, Taylor RW, et al. Melbourne: Melbourne University Press, and Ithaca: Cornell University Press; 1991:141‒179.

S6. Béthoux O, Briggs DEG: **How *Gerarus* lost its head: stem-group Orthoptera and Paraneoptera revisited.** *Syst* *Entom* 2008, **33**:529‒547.

S7. Haug JT, Mayer G, Haug C, Briggs DEG: **A Carboniferous non-onychophoran lobopodian reveals long-term survival of a Cambrian morphotype.** *Curr Biol* 2012, **22**:1673–1675.

S8. Labandeira CC, Santiago-Blay JA: **Abdominal legs of Middle Pennsylvanian *Srokalarva*: early expression of the Distal-less gene in holometabolous insects.** *Geol Soc Am Abstr Prog* 2002, **34**(6):101‒102.

S9. Labandeira CC: **Evidence for an earliest Late Carboniferous divergence time and the early larval ecology and diversification of major Holometabola lineages.** *Entom Am* 2011, **117**:9‒21.

S10. Haug JT, Haug C, Kutschera V, Mayer G, Maas A, Liebau S, et al: **Autofluorescence imaging, an excellent tool for comparative morphology.** *J Microsc* 2011, **244**:259‒272.

S11. Willmann R: **Advances and problems in insect phylogeny**. In Arthropod Relationships. Edited by Fortey RA Thomas RH. London: Chapman and Hall; 1997:270‒279.

S12. Rasnitsyn AP: **Cohors Scarabaeiformes Laicharting, 1781. The holometabolans (= Holometabola Burmeister, 1835, = Endopterygota Sharp, 1899, = Oligoneoptera Martynov, 1938).** In *History of Insects*. Edited by Rasnitsyn AP, Quick, DLJ. Dordrecht: Kluwer; 2002:157‒254.

S13. Grimaldi DA Engel MS: *Evolution of the Insects*. New York: Cambridge University Press; 2005.

S14. Nel A, Roques P, Nel P, Prokop J, Steyer, JS: **The earliest holometabolous insect from the Carboniferous: a “crucial” innovation with delayed success (Insecta Protomeropina Protomeropidae).** *Ann Soc Entom France* 2007, **43**:349‒355.

S15. Nel A, Roques P, Nel P, Prokin AA, Bourgoin T, Prokop J et al: **The earliest known holometabolous insects.** *Nature* 2013, **503**:257‒261.

S16. Walker JD, Geissman JW, Bowring SA Babcock LE: **The Geological Society of America geologic time scale.** *Geol Soc Am Bull* 2013 **125**:259‒272.

S17. Haug JT, Briggs DEG, Haug C: **Morphology and function in the Cambrian Burgess Shale megacheiran arthropod *Leanchoilia superlata* and the application of a descriptive matrix.** *BMC Evol Biol* 2012, **12**:art. 162.

S18. Kristensen NP: **Phylogeny of the endopterygote insects, the most successful lineage of living organisms**. Eur J Entom 1999 **96**: 237–253.

S19. Haug JT, Haug C, Schweigert G, Sombke A: **The evolution of centipede venom claws ‒ Open questions and possible answers.** *Arthro Struc Dev* 2014 **43**:5‒16.

S20. Novokshonov VG: **The first mecopteroids (Insecta: Papilionidea = Mecopteroidea) and the origin of scorpionflies (Panorpida = Mecoptera), with description of a legless eruciform larva from the Lower Permian of Tshekarda.** *Paleont* *J* 2004 **38**:S204‒S213.

**Additional file S5: Figure S1**


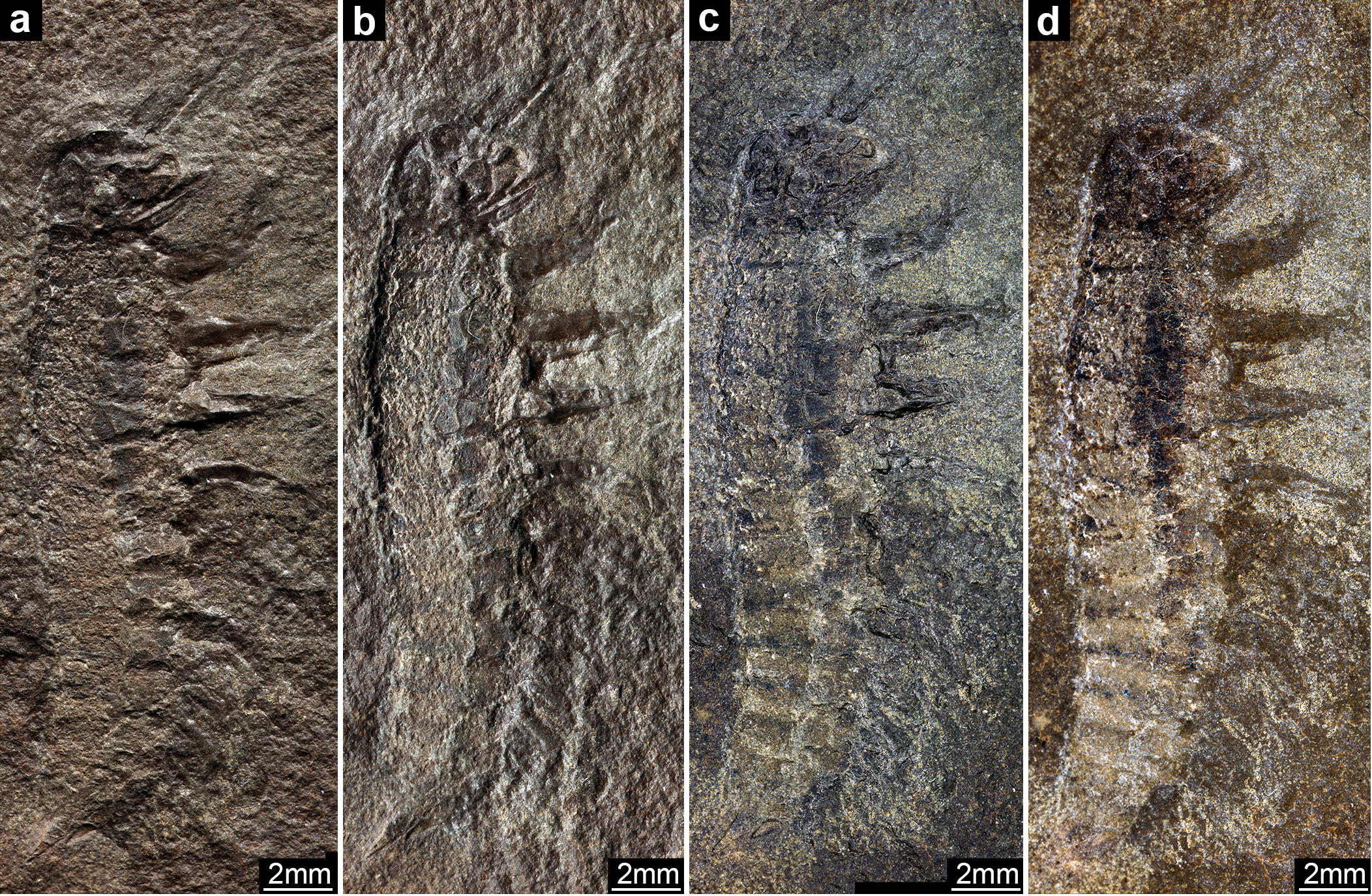


**Figure S1.** Specimen part of MCP-322 imaged by different and variable illumination settings. **(A)** and **(B)**, Unfiltered, low-angle light from two different sources. **(C)**, Unfiltered, reflected light. **(D)**, Cross-polarized light.

**Additional file S6: Figure S2**


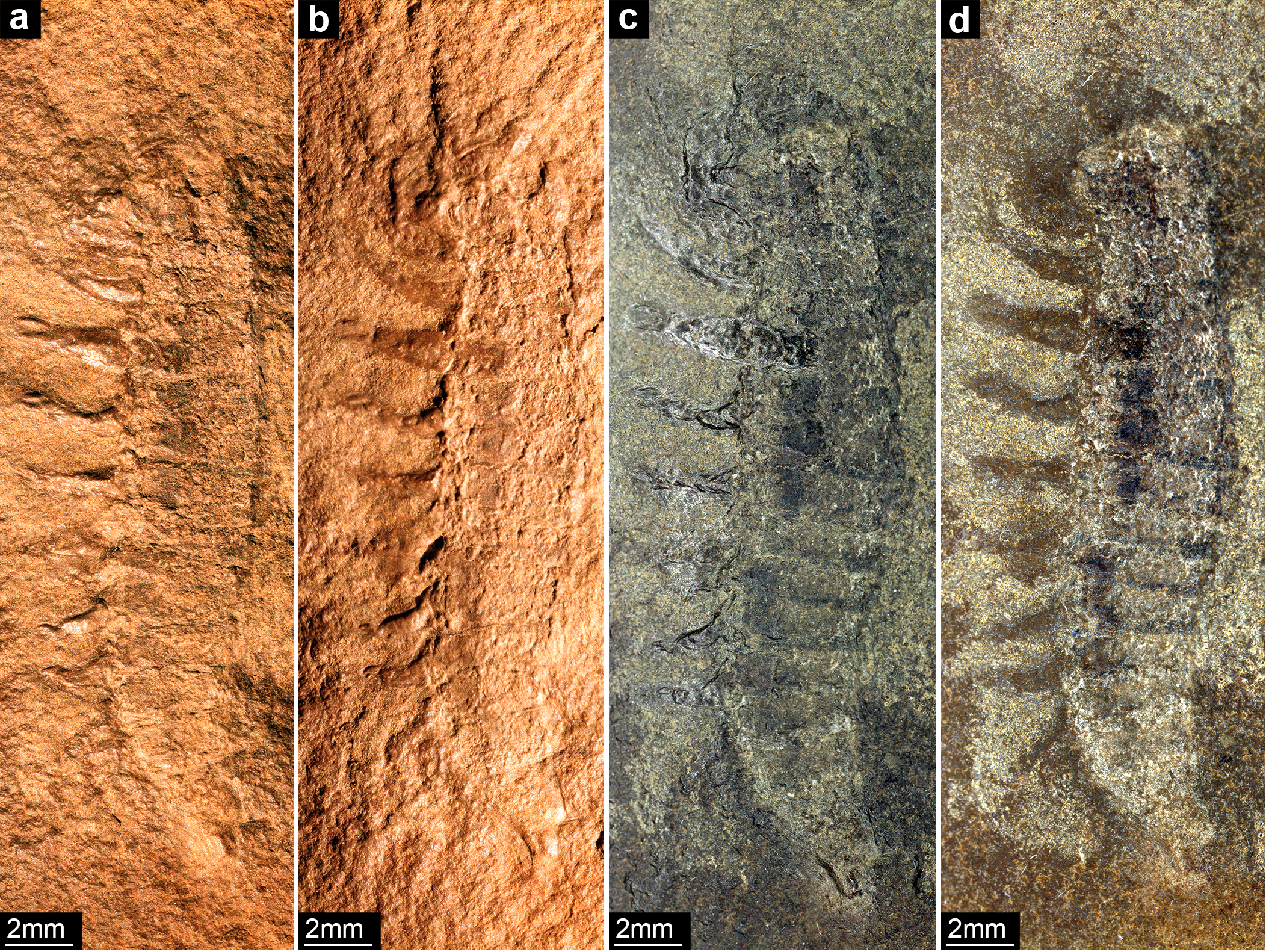


**Figure S2.** Specimen counterpart of MCP-322 imaged by different and variable illumination settings. **(A)** and **(B),** Indicate unfiltered, low-angle light directed from two different sources. **(C)**, Unfiltered, reflected light. **(D)**, Cross-polarized light.

**Additional file S7. Figure S3**


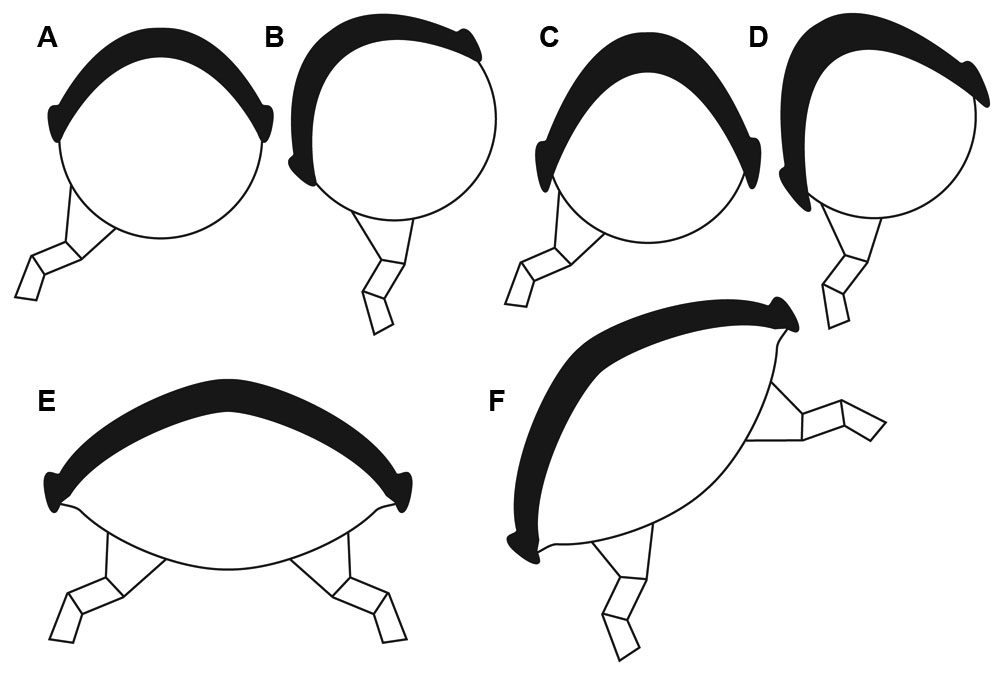


**Figure S3.** Interpretation of matrix embedment angle of *Srokalarva* *berthei*. In **(A)** and **(B)**, a round body cross-section allows the dorsal sclerite (tergite) to reach the midline of lateral body wall. In **(C)** and **(D)**, a round body cross-section, the tergite extends further than the midline of lateral body wall. By contrast, in **(E**) and **(F)**, an oval body cross-section characterized by dorsoventral compression of the body shape, allows for a considerable widening in the dorsal profile of the tergite. (A), (C) and (E) represent a vertical orientation of the dorsal‒ventral body axis, whereas (B), (D) and (F) show a tilted orientation from the left side. The tergites cover the proximal appendicular region only in (F).
